# Glucosidase GANAB drives colorectal cancer progression through Wnt signaling and is modulated by lncRNA FIRRE

**DOI:** 10.3389/fmolb.2026.1820992

**Published:** 2026-08-17

**Authors:** Yajie Wang, Wenjun Li, Qian Hua, Zhengyang Li, Miao Jiang

**Affiliations:** 1 Department of Gastroenterology, Jinshan Hospital, Fudan University, Shanghai, China; 2 Department of Anaesthesiology, Shanghai Public Health Clinical Center, Fudan University, Shanghai, China

**Keywords:** colorectal cancer, GANAB, lncRNA FIRRE, RNA interaction, Wnt/β-catenin signaling

## Abstract

**Background:**

The goal of this study is to investigate the expression pattern of GANAB in colorectal cancer and the role behind it.

**Methods:**

In order to study the expression level of GANAB in the tissue of colorectal cancer, we used quantitative real-time PCR and immunohistochemistry. In the aspect of biological function, we study it through the experimental models. The molecular mechanism is clarified by RNA pull-down and RNA stability assays. Student's t-tests and one-way ANOVA are used for data analysis.

**Results:**

Analysis of tissue microarrays and Colorectal cancer (CRC) cell lines revealed elevated GANAB expression in tumors compared to normal counterparts. Increased GANAB levels were linked to enhanced cell migration, invasion, and proliferation, and correlated with metastatic potential and unfavorable patient outcomes. Functional enrichment studies indicated that GANAB-associated genes participate in the Wnt/β-catenin signaling pathway. Immunohistochemistry confirmed positive correlations between GANAB and both c-Myc and β-catenin. The oncogenic function of GANAB was shown to rely on Wnt pathway activation. Additionally, lncRNA FIRRE, known to be oncogenic in CRC, was found to physically interact with GANAB mRNA *via* RNA pull-down assays. FIRRE enhances GANAB mRNA stability through direct binding.

**Conclusion:**

Our study demonstrates that GANAB exerts oncogenic effects in CRC by binding directly to lncRNA FIRRE, thereby stabilizing GANAB mRNA and activating the Wnt/β-catenin pathway through nuclear accumulation of β-catenin.

## 1 Introduction

Colorectal cancer (CRC) represents a prevalent global health challenge due to its worldwide incidence (Siegel et al., 2020). According to estimations, there are more than 380,000 new CRC cases in China each year (Wu et al., 2019). Further elucidation of CRC pathogenesis is required to devise better treatment strategies.

Cancer-related gene activity defies straightforward regulations; it unfolds as a complex orchestration across transcriptional and post-transcriptional stages. The latter determines RNA splicing, stability, subcellular localization, and translation (Jankowsky and Harris, 2015). In this complex regulation, coding and noncoding RNA species interact with their cognate RNA-binding proteins to finely modulate cellular functions (Cui et al., 2025). Recently, the complex interplay between lncRNAs and mRNAs has been revealed as a powerful means to finely tune oncogenic biological networks (Statello et al., 2021). For example, long non-coding RNAs may interact with their corresponding mRNA transcripts, affecting the stability of mRNA or the production of protein, which will interfere with the process of cell proliferation and metastasis (Jankowsky and Harris, 2015).

The Wnt/β-catenin signaling pathway is generally considered as a major driving factor for the development of colorectal cancer. Yet beyond its traditional role in turning genes on and off, fresh evidence points to a two-way street where Wnt signaling converses with post-transcriptional RNA handling. Key components of the Wnt pathway are subject to regulation via alternative splicing and mRNA stability control, forming feedback loops that potentiate oncogenic signaling (Bordonaro, 2013). However, the upstream regulators that modulate this post-transcriptional dimension of Wnt signaling in CRC remain incompletely understood.

In our initial bioinformatic analysis, we found that GANAB (glucosidase II alpha subunit) was upregulated in CRC tissues. Although the role of GANAB in protein glycosylation in the endoplasmic reticulum is well known (Khaodee et al., 2019; Porath et al., 2016; Su et al., 2023; Lin et al., 2022a), its role and regulatory mechanism through post-transcriptional modifications in CRC are unknown. In another study, we have previously characterized lncRNA FIRRE, an evolutionarily conserved lncRNA located on the X chromosome (Hacisuleyman et al., 2014), as an oncogenic factor in CRC (Wang et al., 2022). Given the paradigm that lncRNAs regulate the fate of mRNAs (Statello et al., 2021), we tested the hypothesis that FIRRE may interact with GANAB.

We determined the expression of GANAB in CRC and explored the functional effects of GANAB on the malignant phenotype of CRC and the underlying molecular mechanism, especially the Wnt signaling and the regulation of lncRNA FIRRE.

## 2 Methods

### 2.1 Patient samples and tissue microarray construction

A tissue microarray comprising 180 CRC samples (94 tumors, 86 matched adjacent tissues) was obtained from Shanghai OutDo Biotech. Pathological diagnosis was confirmed by two expert pathologists. Clinical and pathological data were recorded, and staging followed the AJCC eighth edition TNM classification.

### 2.2 Cell culture

CRC cell lines (SW480, HCT-8, LOVO, HT29) and normal colonic epithelial cells (NCM460) were acquired from KeyGEN BioTECH. SW480, HCT-8, HT29 and NCM460 cells were cultured in DMEM. LOVO cells were maintained in RPMI 1640. All medium was supplemented with 10% fetal bovine serum and 1% penicillin-streptomycin (100 U/mL penicillin and 100 μg/mL streptomycin) at 37 °C in a 5% CO_2_.

### 2.3 Immunohistochemistry

The immunohistochemical analysis was carried out following established procedures (Thermo Scientific). Immunohistochemistry was performed using an anti-GANAB primary antibody (Abcam) diluted 1:500 in Proteintech IHC antibody diluent, followed by an HRP-conjugated secondary antibody. Signal was detected with a DAB substrate kit (Thermo Scientific). Two independent pathologists, who were blinded to the patients’ clinical backgrounds, evaluated both the depth of staining (ranging from 0 to 3) and the proportion of cells showing positive results (0%–100%). IHC score: intensity 0–3,positive rate 0%–100%,final score = intensity × percentage,cut-off value = 170。.

### 2.4 TCGA data analysis

GANAB expression and clinical correlations were analyzed using TCGA datasets. Pathway enrichment was evaluated *via* GSEA.

### 2.5 Western blot and qRT-PCR

For Western blotting, proteins extracted from cells or tissues were separated by SDS-PAGE and transferred onto PVDF membranes (Millipore, United States). After blocking with 5% non-fat milk for 1 h at room temperature, the membranes were incubated overnight at 4 °C with primary antibodies against GANAB (Abcam, diluted 1:1,000) and GAPDH (Proteintech, diluted 1:5,000). Following three washes, the membranes were incubated with HRP-conjugated secondary antibody (diluted 1:2000) for 1 h at room temperature. Immunoreactive bands were visualized using an ECL system. For RNA extraction and qRT-PCR, total RNA was isolated using TRIzol reagent (Invitrogen, United States). cDNA was synthesized using a Takara RT-PCR kit (Takara, Japan), and qRT-PCR was performed with SYBR Premix Ex Taq (Takara, Japan) on Applied Biosystems 7,500 system. GAPDH was used as the endogenous control. Primer sequences are provided in Supplementary Table S1. All experiments were performed with three independent biological replicates and three technical replicates.

### 2.6 Plasmid construction and transfection

The FIRRE plasmids were constructed for detecting the function of FIRRE on the GANAB RNA level. The pUC19 vector (Beyotime Biotechnology, Shanghai, China) acted as an empty vector and then were applied to combine with the wild-type and mutant FIRRE separately. ASO targeting FIRRE were designed by RiboBIO (Guangzhou, China) and transfected using Lipofectamine 3,000. Sequences are provided in Supplementary Table S2.

### 2.7 Proliferation, migration, and invasion assays

Cell proliferation was measured using the CCK-8 assay (Dojindo). Cells were seeded at 1.0 × 10^4^ cells/well in 96-well plates, and absorbance at 450 nm was recorded every 12 h. For migration and invasion assays, Transwell chambers (8-μm pores, Corning) were used. For invasion, the upper chamber was pre-coated with Matrigel (Corning). Cells (5 × 10^4^) in serum-free medium were added to the upper chamber, and medium with 20% FBS was placed in the lower chamber. After 48 h, cells on the lower side were fixed with 4% paraformaldehyde, stained with crystal violet, and counted in five random fields per well.

### 2.8 Subcellular fractionation

Nuclear and cytoplasmic extracts were prepared through a commercial kit (Invent, United States). Lamin B1 and GAPDH were utilized as nuclear and total controls.

### 2.9 RNA interaction prediction and validation

Biotin-labeled FIRRE and control GAPDH RNAs were transcribed *in vitro* using the MEGAscript T7 Kit (Life Technology) and RiboMAX™ RNA Production System (Promega), with biotin-14-UTP (Thermo Scientific). The biotinylated RNAs were incubated with CRC cell lysates, followed by addition of streptavidin magnetic beads (Thermo Scientific). After washing, bound RNAs were extracted with TRIzol, and GANAB mRNA enrichment was measured by qRT-PCR as fold change relative to GAPDH control.

### 2.10 Wnt pathway activation

We used CHIR-99021 (MedChemExpress, China) to activate Wnt signaling pathway in the reprogrammed HT29 and LOVO cells. The CHIR-99021 was co-cultured with the reprogrammed HT29 and LOVO cells at the concentration of 5 nmol/L for 24 h.

### 2.11 Statistical analysis

We utilized GraphPad Prism and SPSS 22.0 for our statistical analyses. For continuous variables, we employed t-tests and ANOVA to compare means. Categorical data were examined using either Pearson’s or Fisher’s exact test, depending on the sample size. To assess survival outcomes, we applied Kaplan-Meier curves and conducted Cox regression analysis. Statistical significance was determined at p < 0.05.

## 3 Result

### 3.1 GANAB is overexpressed in CRC and correlates with aggressive clinicopathological features

**FIGURE 1:**
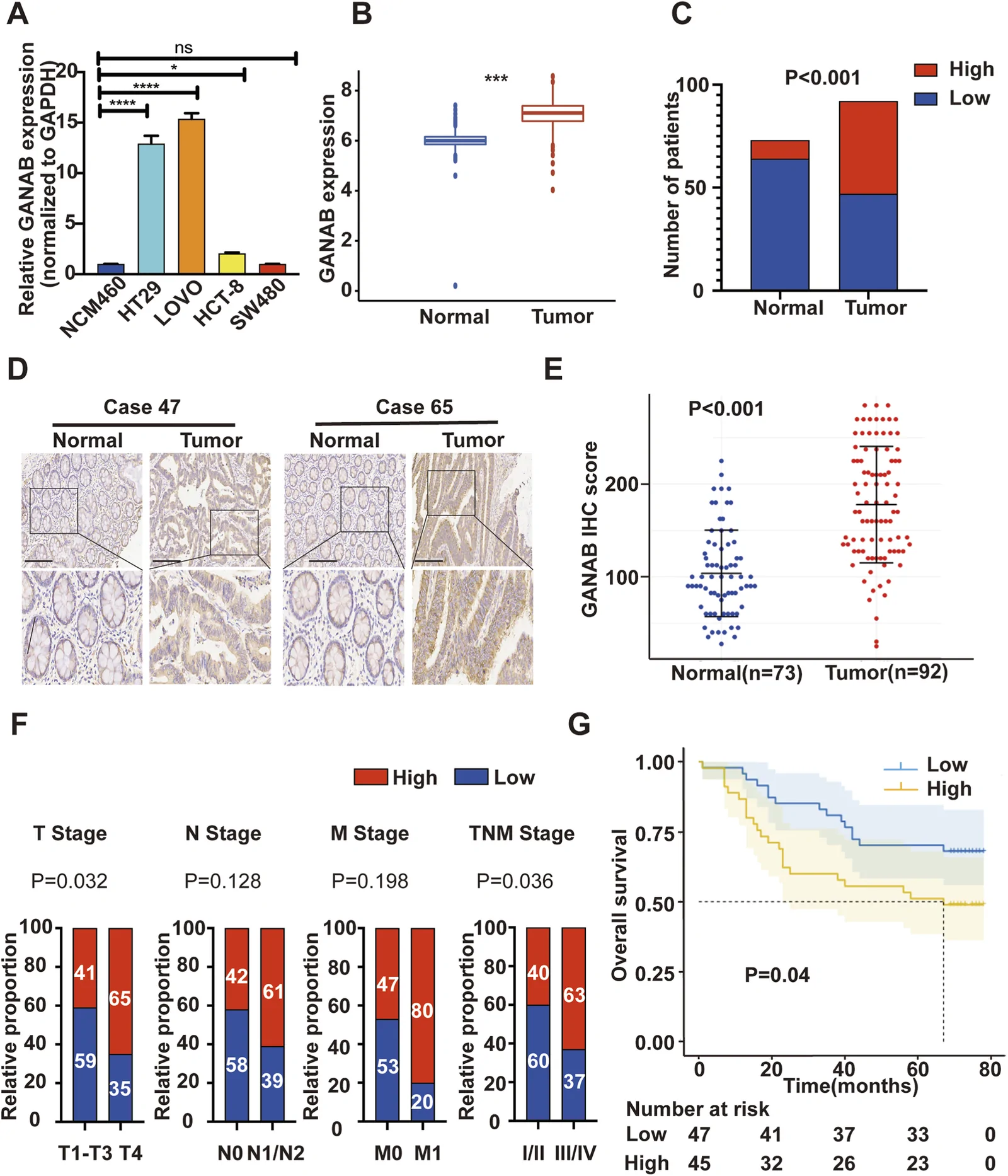
GANAB is overexpressed in colorectal cancer and correlates with aggressive clinicopathological features. **(A)** Quantitative real-time PCR (qRT-PCR) analysis of GANAB mRNA levels in the normal colonic epithelial cell line NCM460 and four colorectal cancer (CRC) cell lines (SW480, HCT-8, LOVO, HT29). Data are presented as mean ± SD from three independent experiments. **(B)** Analysis of GANAB mRNA expression in CRC tissues and normal colon mucosa from The Cancer Genome Atlas (TCGA) dataset. **(C)** Western blot showing GANAB protein expression in paired clinical specimens (tumor vs. adjacent non-tumor tissues from 73 normal and 92 cancer cases). GAPDH served as a loading control. **(D)** Representative immunohistochemical staining images of GANAB in paired CRC tissues (tumor, T; adjacent non-tumor, N) from two representative patients (case 47 and case 65) on the tissue microarray. Scale bar = 200 μm. **(E)** Semi-quantitative IHC scores of GANAB expression in tumor tissues compared with matched non-tumor tissues. Each dot represents one patient. **(F)** Bar graph depicting the relative proportion of CRC patients with high or low GANAB expression across T, N, M, and TNM stages (analyzed by Chi-square test). **(G)** Kaplan–Meier overall survival (OS) curves for CRC patients stratified by high vs. low GANAB expression. Log-rank test was used for comparison. Statistical significance: *p < 0.01, ***p < 0.001, ****p < 0.0001; ns, not significant.

Following our analysis, we examined the mRNA expression patterns of GANAB across a panel of colorectal cancer cell lines, including SW480, HCT-8, LOVO, and HT29, using NCM460 as a control for normal colonic epithelial tissue. The quantitative PCR data painted a clear picture, revealing that GANAB expression was significantly elevated in the majority of the CRC cell lines we investigated (Figure 1A). Analyzing the public transcriptomic data from TCGA further confirmed this, which demonstrated that GANAB mRNA was overexpressed in CRC tissues compared with normal colon mucosa (Figure 1B).

This was further confirmed by immunohistochemical (IHC) staining of tissue microarrays. The result showed that GANAB was overexpressed in the majority of tumor tissues compared with adjacent non-tumor tissues (Figure 1C). GANAB protein mainly localized to the cytoplasm and was more strongly stained in tumor tissues than in matched normal tissues (Figure 1D). Semiquantitative scoring of the IHC intensity further confirmed the significant increase of GANAB expression in tumor tissues (Figure 1E).

To gauge the clinical importance of GANAB, we divided the patients into two groups—those with high and those with low expression—based on their immunohistochemical assessments. Our correlation analysis with clinicopathological characteristics (detailed in Table 1) demonstrated that heightened GANAB expression was strongly linked to larger tumor dimensions (P = 0.005), more profound tissue invasion (T stage, P = 0.032), and later-stage TNM classification (P = 0.036). But we found no meaningful connections between GANAB levels and patient gender, age, histological grading, or the presence of nodal (N) or distant (M) metastases within our study population. The distribution of high GANAB expression across T stages indicated its potential role in enhancing local invasiveness (Figure 1F). As it turned out, the Kaplan-Meier survival curves painted a pretty clear picture: patients with higher GANAB expression levels were sitting ducks when it came to overall survival, lagging far behind those with lower expression when the chips were down (Figure 1G). Furthermore, univariate Cox regression analysis positioned GANAB expression as a *bona fide* harbinger of unfavorable clinical outcomes; however, multivariate analysis suggested it may not serve as an entirely independent prognostic indicator in our cohort (Supplementary Tables S3, 4).

**TABLE 1:** Correlation between GANAB expression and clinicopathological characteristics.

Variables		GANAB expression		Total	p value
		Low IHC Score* ≤170%	High IHC Score* >170%		
Sex	Male	25	21	46	0.677
	Female	22	24	46	
Age (year)	≤65	27	21	48	0.404
	>65	20	24	44	
Grade	I/II	42	36	78	0.254
	III/IV	5	9	14	
Tumor size	≤5 cm	32	15	47	0.005*
	>5 cm	14	25	39	
T stage	T1–T3	36	25	61	0.032*
	T4	11	20	31	
N stage	N0	34	25	59	0.128
	N1/N2	13	20	33	
M stage	M0	46	41	87	0.198
	M1	1	4	5	
TNM stage	I/II	34	23	57	0.036*
	III/IV	13	22	35	

*IHC score = staining intensity (0, 0.5, 1, 2, 3) × percentage of positive cells (0−100%). Score ≤170%: low expression; >170%: high expression; Bold values indicate statistical significance (P < 0.05).

### 3.2 GANAB promotes CRC cell proliferation and invasion

**FIGURE 2:**
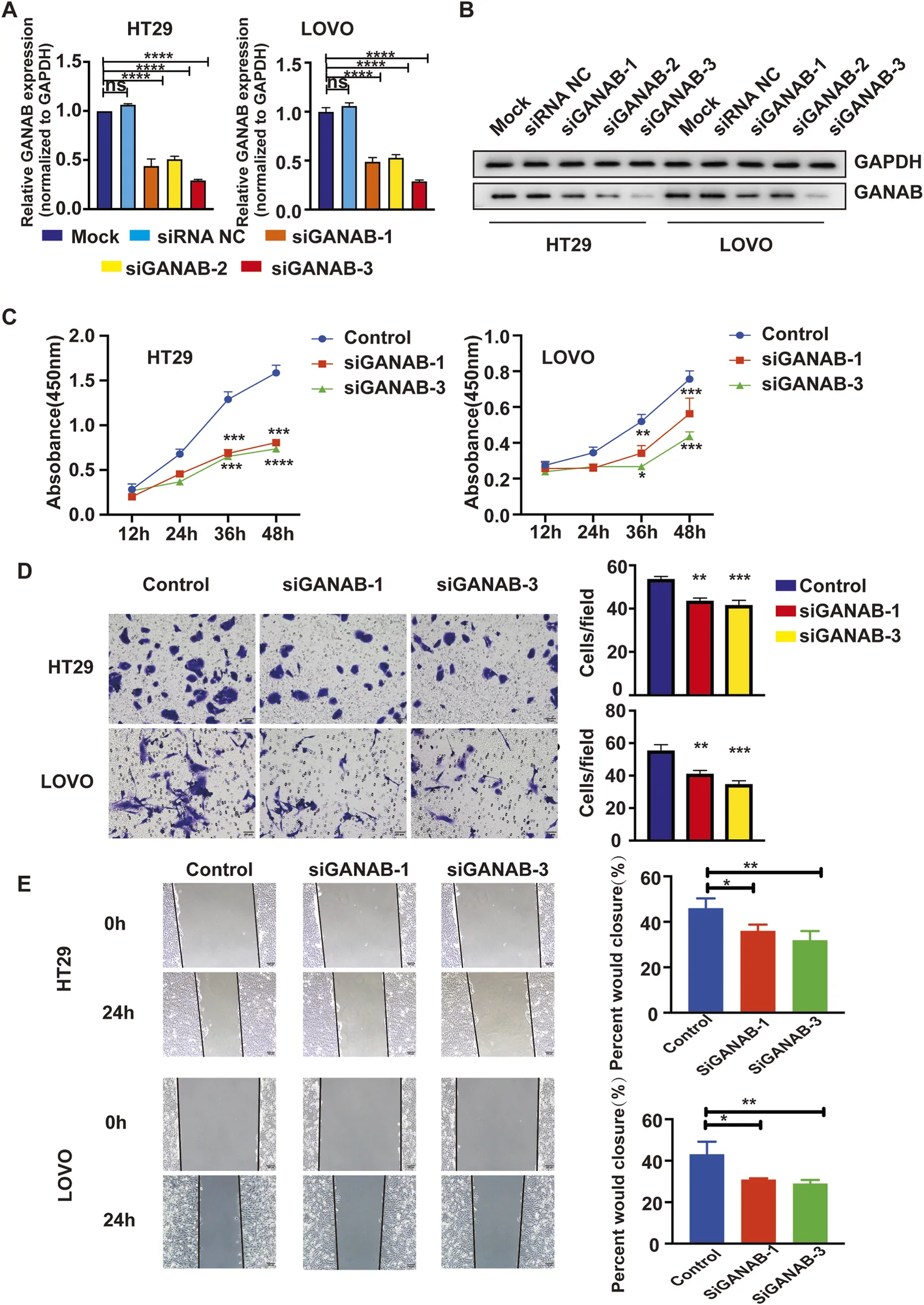
GANAB promotes CRC cell proliferation and invasion. **(A,B)** GANAB expression was assessed by qRT-PCR **(A)** and Western blotting **(B)** in HT29 and LOVO cells, which transfected with control siRNA (si-NC) or two independent GANAB-targeting siRNAs (si-GANAB#1, #3). Data are presented as mean ± SD from three independent experiments. **(C)** Cell proliferation was measured by CCK-8 assay in HT29 and LOVO cells after GANAB knockdown. Absorbance at 450 nm was tested at the designed time points. Data are shown as mean ± SD of triplicate wells. **(D)** The invasive capability of cells was evaluated using Matrigel-coated Transwell chambers. Representative images (left) and quantitative analysis (right) of invaded cells after 48 h are shown. Scale bar = 50 μm. Data are mean ± SD from three independent experiments. **(E)** Cell migration was evaluated by wound healing assay. Representative images at 0 and 24 h (left) and quantitative analysis of wound closure rate (right) are presented. Data are mean ± SD from three independent experiments. Statistical significance: *p < 0.01, ***p < 0.001, ****p < 0.0001.

Given that high GANAB expression was correlated with aggressive tumor features, we further examined its functional role by loss-of-function assays. Specific siRNAs (siRNA1 and siRNA3) effectively silenced GANAB at the mRNA and protein levels in HT29 and LOVO cells, as shown by qRT-PCR and WB (Figures 2A,B).

CCK-8 proliferation assays demonstrated that GANAB depletion significantly slowed HT29 and LOVO cell growth over 72 h (Figure 2C). Transwell assays evaluated possible effects on metastatic potential. Silencing GANAB led to a significant decrease in the number of cells that migrated through the uncoated membranes and breached the Matrigel-coated barriers. (Figure 2D). Wound healing assays were conducted under serum-free conditions to avoid proliferation interference in measuring migration. Consistent with the Transwell results, GANAB knockdown also significantly inhibited the CRC cells’ migratory ability, as evidenced by slower wound closure (Figure 2E). In contrast, Supplementary Figure S1 showed that overexpression of GANAB reversed the above results in terms of proliferative, migratory, and invasive abilities.

### 3.3 The oncogenic function of GANAB is mediated through activation of the Wnt/β-catenin pathway

**FIGURE 3:**
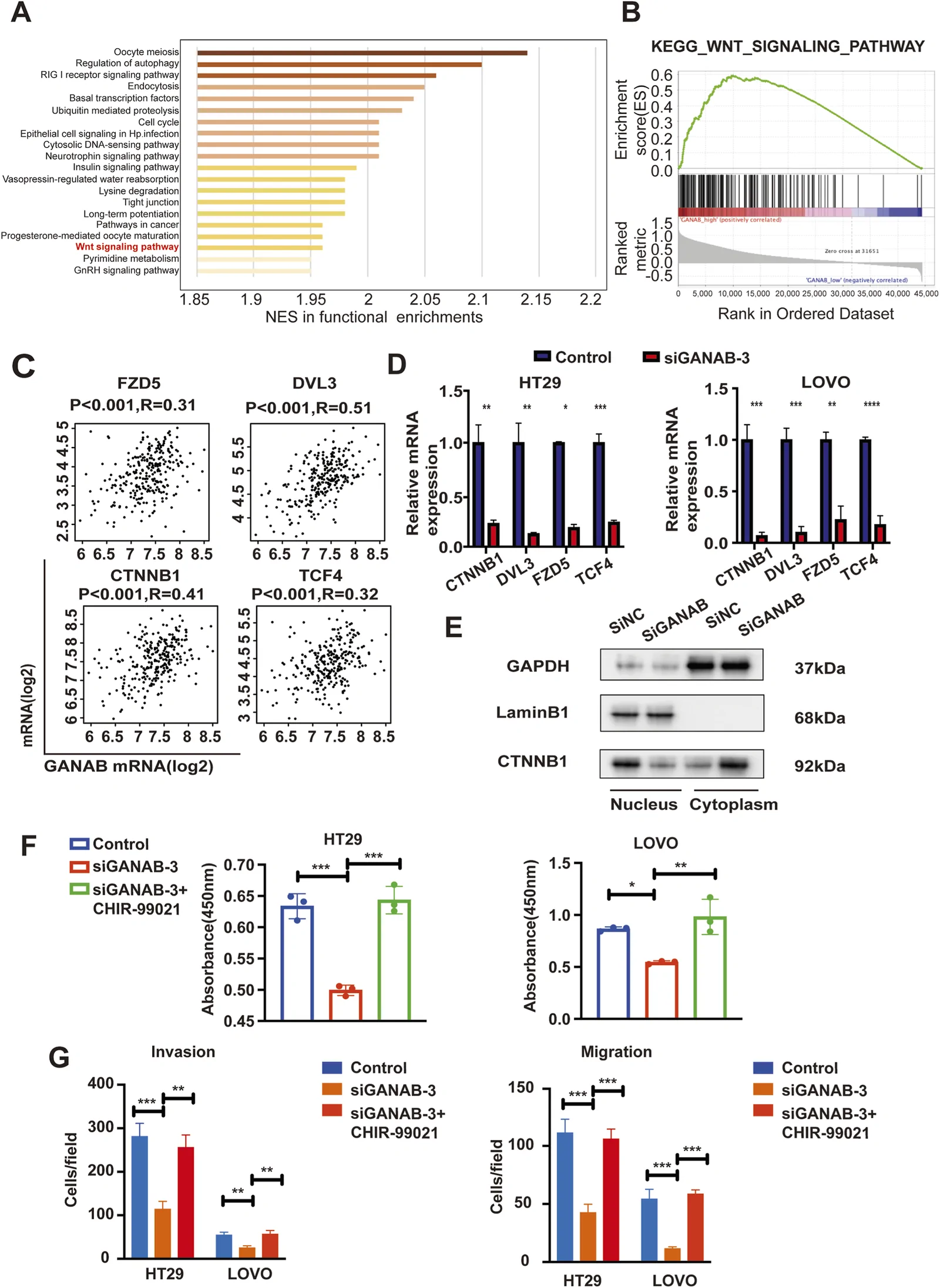
The oncogenic function of GANAB is mediated through activation of the Wnt/β-catenin pathway. **(A)** GSEA was performed on RNA-seq data from the TCGA-COAD cohort, stratified by high versus low GANAB expression. The top 20 significantly enriched KEGG pathways are ranked by NES (NES = normalized enrichment score). **(B)** GSEA plot demonstrating significant enrichment of the “Wnt signaling pathway” gene set in tumors with high GANAB expression **(C)** Correlation analysis between GANAB mRNA levels and the expression of core Wnt pathway genes (FZD5, DVL3, CTNNB1, TCF4) within the TCGA-COAD dataset. Pearson’s correlation coefficient (r) and p-value are indicated for each gene. **(D)** qRT-PCR analysis validating the mRNA levels of FZD5, DVL3, CTNNB1, and TCF4 in HT29 and LOVO cells transfected with control siRNA (si-NC) or GANAB-targeting siRNA (si-GANAB). Data are presented as mean ± SD from three independent experiments. **(E)** qRT-PCR analysis validating the mRNA levels of cyclinD1 and C-myc in LOVO cells transfected with control siRNA (si-NC) or GANAB-targeting siRNA (si-GANAB). Data are presented as mean ± SD from three independent experiments. **(F)**Western blot analysis of nuclear β-catenin protein levels in HT29 and LOVO cells following GANAB knockdown. Lamin B1 and GAPDH serve as nuclear and total loading controls, respectively **(G)** Rescue of proliferation by Wnt pathway activation. CCK-8 assay was performed on GANAB-silenced cells treated with or without the Wnt agonist CHIR-99021 (5 nmol/L). Data (mean ± SD) show absorbance at 450 nm. **(G)** Rescue of migration and invasion. Transwell migration (uncoated) and invasion (Matrigel-coated) assays were conducted on GANAB-silenced cells treated with CHIR-99021 (5 nmol/L). The bar graph shows the mean ± SD number of cells. All quantitative data are expressed as mean ± SD of three biological replicates. Statistical significance: *p < 0.01, ***p < 0.001, ****p < 0.0001.

To get to the bottom of GANAB’s molecular function, we ran a Gene Set Enrichment Analysis (GSEA) using TCGA-COAD data, stratifying samples based on GANAB expression levels. Our findings reveal that the Wnt/β-catenin signaling pathway gene set shows significant upregulation in GANAB-high tumors, as clearly illustrated in Figures 3A,B. Wnt/β-catenin signaling pathway is an important regulator of many biological functions and also plays a great role in the development of cancer (Lin et al., 2022a). Further study with TCGA data showed that the level of GANAB mRNA was positively correlated with the expression of key molecules in Wnt pathway, such as receptor FZD5, signal transduction protein DVL3, core effector molecule CTNNNB1 (β-catenin) and transcription factor TCF4 (Figure 3C).

We experimentally validated this bioinformatic prediction. qRT-PCR analysis confirmed that knockdown of GANAB in HT29 and LOVO cells led to a coordinated downregulation of FZD5, DVL3, CTNNB1, TCF4, CYCLIND1 and C-MYC mRNA (Figures 3D,E). Since nuclear accumulation of β-catenin is a mark of canonical Wnt pathway activation, we tested its subcellular localization following GANAB silencing. Western blot analysis of nuclear fractions demonstrated a clear reduction in nuclear β-catenin protein upon GANAB knockdown (Figure 3F).

To establish a causal link, we performed rescue experiments using the pharmacological Wnt pathway activator CHIR-99021. Treatment with CHIR-99021 effectively reversed the anti-proliferative effect (Figure 3G) and restored the migratory and invasive capabilities (Figure 3H) that were suppressed by GANAB depletion. Collectively, these results demonstrate that GANAB facilitates tumor growth primarily through Wnt/β-catenin pathway activation.

### 3.4 The oncogenic lncRNA FIRRE directly interacts with and stabilizes GANAB mRNA

**FIGURE 4:**
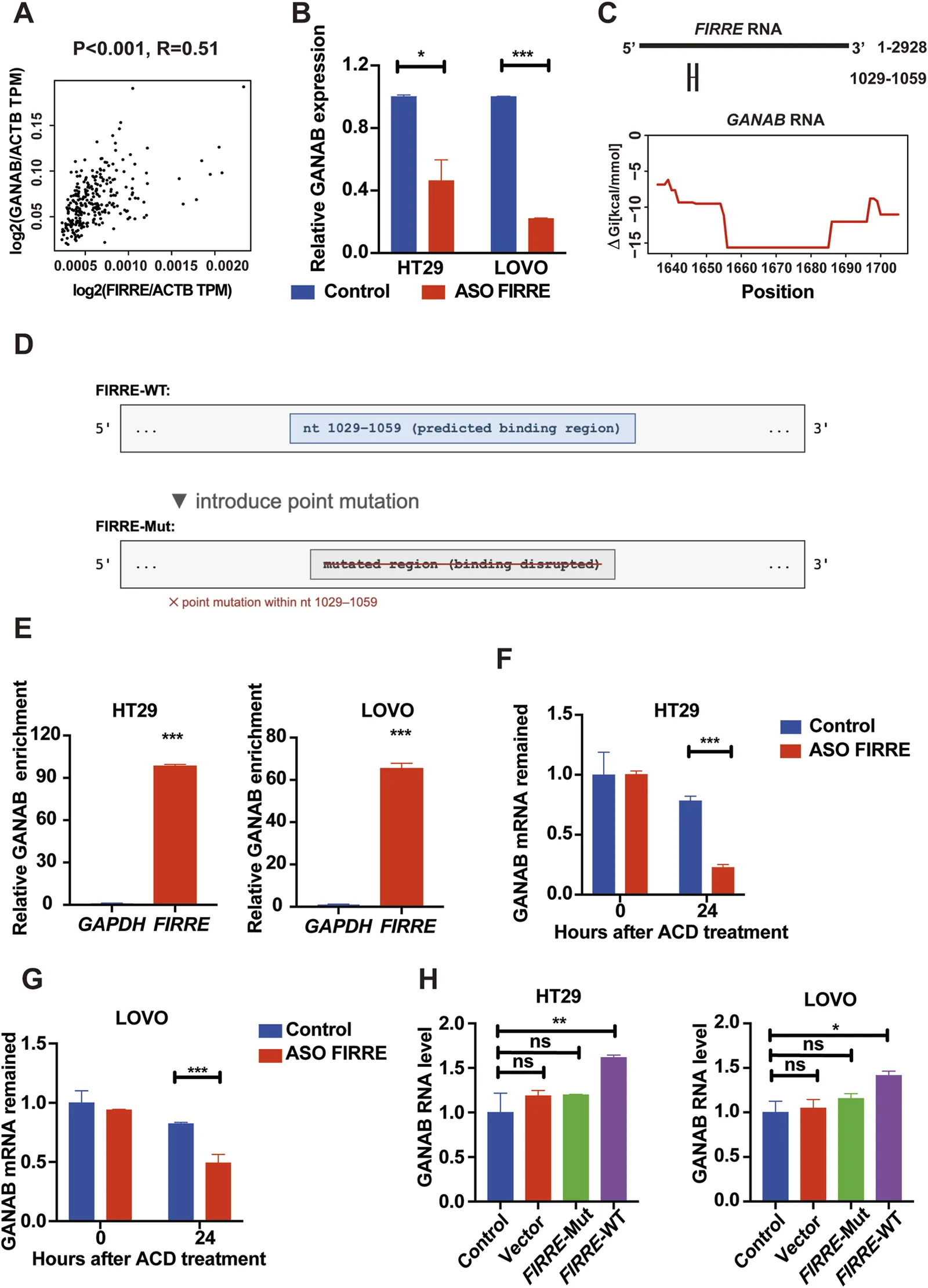
LncRNA FIRRE directly binds to and stabilizes GANAB mRNA. **(A)** Correlation analysis between FIRRE and GANAB mRNA expression in colorectal cancer tissues, showing a significant positive association (Pearson’s correlation). **(B)** GANAB mRNA levels were measured by qRT-PCR in HT29 and LOVO cells transfected with antisense oligonucleotides targeting FIRRE (ASO-FIRRE) or a negative control (ASO-NC). Data are presented as mean ± SD from three independent experiments. **(C)** In silico prediction of RNA–RNA interaction between FIRRE and GANAB mRNA using the online tools Starbase 2.0 and RNAup. The upper panel indicates the predicted binding region in FIRRE (nucleotides 1,029–1,059), and the lower panel shows the calculated interaction free energy (ΔGi) profile across the GANAB sequence. **(D)** Schematic of wild-type FIRRE (FIRRE-WT) showing the predicted GANAB-binding region (nucleotides 1,029–1,059) and mutant FIRRE (FIRRE-Mut) with a point mutation introduced in this region. **(E)** RNA pull-down assay followed by qRT-PCR validation. Biotin-labeled FIRRE or GAPDH (negative control) transcripts were incubated with whole-cell lysates. The specifically bound GANAB mRNA was quantified by qRT-PCR and is shown as fold enrichment relative to the GAPDH control. **(F,G)** mRNA stability assay. Control (ASO-NC) or FIRRE-depleted (ASO-FIRRE) HT29 **(F)** and LOVO **(G)** cells were coincubated with actinomycin D (Act.D, 5 μg/mL). The remaining GANAB mRNA levels at the indicated time points were quantified by qRT-PCR and normalized to the 0-h value. **(H)** Functional validation of the binding site. HT29 and LOVO cells were co-transfected with empty vector, wild-type FIRRE (FIRRE-WT), or a mutant FIRRE lacking the predicted binding site (FIRRE-Mut). GANAB mRNA levels were assessed by qRT-PCR. Data are shown as mean ± SD of three independent experiments. Statistical significance: *P < 0.05, **P < 0.01, ***P < 0.001; ns, not significant.

We previously identified lncRNA FIRRE as an oncogenic driver in CRC (Wang et al., 2022). Here, analysis of TCGA data indicated a vital positive correlation in FIRRE and GANAB mRNA levels (Figure 4A). To test for a functional interaction, FIRRE expression was knocked down in HT29 and LOVO cells using antisense oligonucleotides (ASOs). This depletion resulted in a concomitant reduction in GANAB mRNA (Figure 4B), suggesting FIRRE may regulate GANAB expression.

Given that lncRNAs can modulate target mRNA stability through direct base-pairing interactions (Zhang et al., 2018; Clevers and Nusse, 2012), we employed bioinformatic tools (Starbase 2.0 and RNAup) to predict potential binding between FIRRE and GANAB mRNA. A putative interaction site was identified within the FIRRE sequence (nucleotides 1,029–1,059) (Figure 4C). Figure 4D showed wild-type FIRRE (FIRRE-WT) with the predicted binding region (nt 1,029–1,059) and mutant FIRRE (FIRRE-Mut) with a point mutation introduced in this region. An RNA pull-down assay using biotin-labeled FIRRE transcript confirmed a specific physical interaction, as GANAB mRNA was significantly enriched in the FIRRE pull-down fraction compared to the control GAPDH RNA pull-down (Figure 4E). The efficiency of biotin labeled FIRRE pull down was confirmed by qRT PCR for FIRRE, showing comparable recovery across all samples (data not shown).

To determine if this interaction affects mRNA stability, we treated control and FIRRE-depleted cells with the transcription inhibitor actinomycin D. The decay rate of GANAB mRNA was significantly accelerated in cells lacking FIRRE, indicating that FIRRE binding enhances the stability of GANAB transcripts (Figures 4F,G). Finally, to validate the specificity of the predicted binding site, a point mutation was introduced into FIRRE (FIRRE-Mut). While overexpression of wild-type FIRRE (FIRRE-WT) increased GANAB mRNA levels, the mutant version failed to do so (Figure 4H), confirming that the direct interaction is required for FIRRE-mediated regulation.

### 3.5 FIRRE influences Wnt/β-catenin signaling activity in a GANAB-dependent manner

**FIGURE 5:**
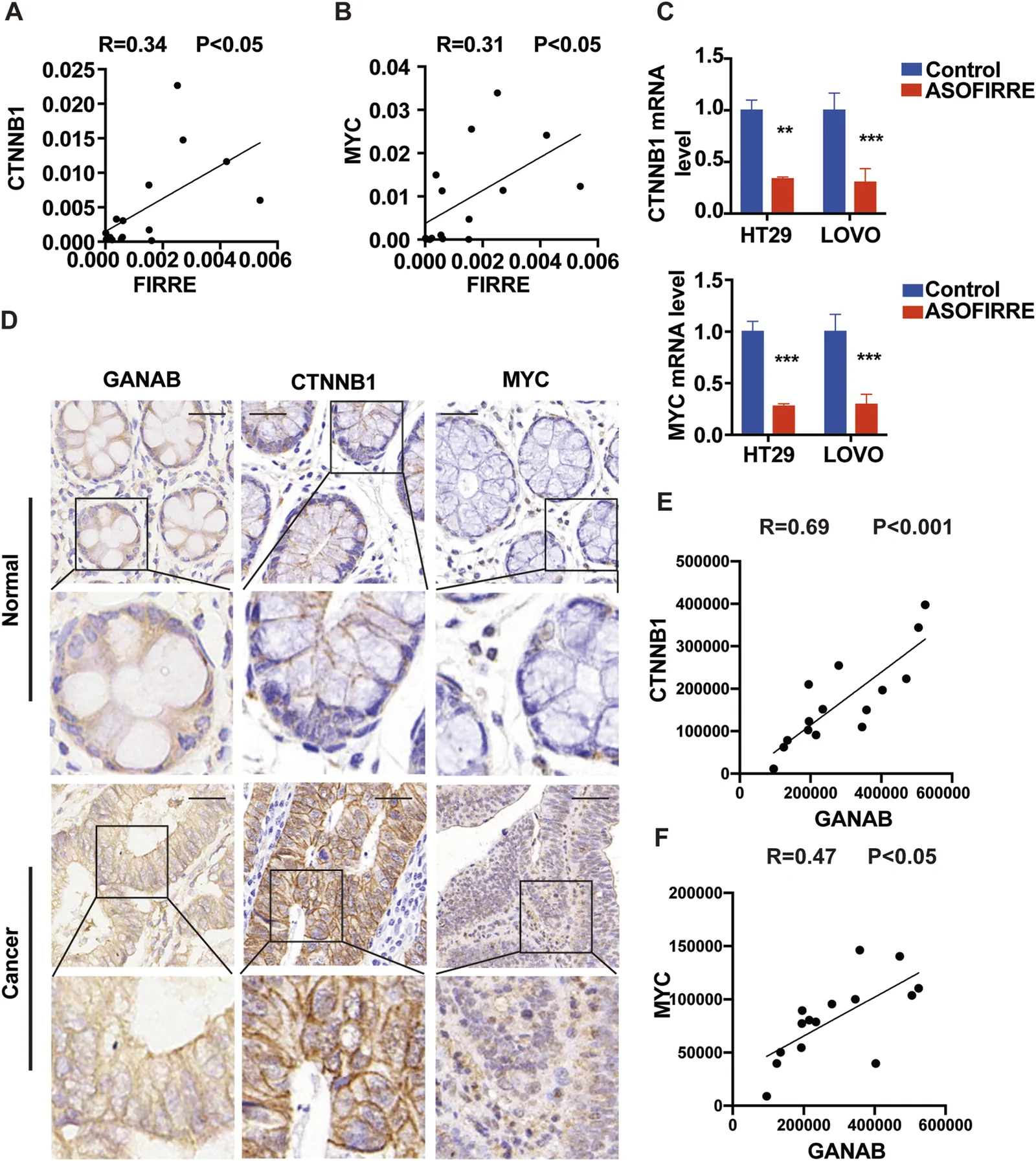
FIRRE modulates the Wnt/β-catenin pathway through GANAB. **(A,B)** Pearson correlation analysis showing a significant positive association between FIRRE mRNA levels and the expression of **(A)** CTNNB1 (β-catenin) and **(B)** MYC (c-Myc) in a cohort of 15 clinical CRC samples. **(C)** qRT-PCR analysis of CTNNB1 and MYC mRNA levels in HT29 and LOVO cells transfected with control ASO (ASO-NC) or FIRRE-targeting ASO (ASO-FIRRE). Data are presented as mean ± SD. **(D)** Representative immunohistochemical (IHC) staining images of serial sections from CRC tissues showing the protein expression of GANAB, β-catenin (CTNNB1), and c-Myc (MYC) in tumor and adjacent non-tumor areas. Scale bar = 200 μm. **(E,F)** Scatter plots and correlation analysis of IHC scores demonstrating a positive correlation between GANAB protein expression and the levels of **(E)** β-catenin and **(F)** c-Myc in CRC tissues. Scale bar in insets = 50 μm. All quantitative data are expressed as mean ± SD. Statistical significance: **p < 0.01, ***p < 0.001.

Based on the established role of GANAB in activating Wnt/β-catenin signaling (Results Section 3) and its regulation by FIRRE, we investigated whether FIRRE influences the Wnt pathway. In a cohort of clinical CRC specimens, FIRRE expression levels showed vital positive correlations with the mRNA levels of the core pathway component CTNNB1 (β-catenin) and the canonical Wnt target gene MYC (c-Myc) (Figures 5A,B). Functionally, knockdown of FIRRE in CRC cell lines led to a reduction in both CTNNB1 and MYC levels (Figure 5C).

At the protein level, immunohistochemical analysis of serial tumor sections revealed a coordinated expression pattern. Specimens displaying elevated GANAB levels demonstrated a robust immune response against both β-catenin and c-Myc (Figure 5D). Quantitative assessment of immunohistochemical scores revealed a significant positive correlation between GANAB protein concentration and the levels of both β-catenin and c-Myc (Figures 5E,F). In short, these results indicate that FIRRE promotes the expression of GANAB protein by stabilizing its mRNA, thus enhancing the Wnt/β-catenin signaling pathway in colorectal cancer.

## 4 Discussion

Our research reveals a new FIRRE/GANAB/Wnt/β-catenin signal axis, which promotes the development of CRC through post-transcriptional regulation mechanism. While earlier research has pinpointed GANAB’s involvement in a multitude of ailments, our research delves into GANAB’s specific biological function within CRC and identifies potential molecular targets that GANAB could influence. Firstly, our investigation into the spatial distribution and expression patterns of GANAB within CRC revealed significantly higher cytoplasmic concentrations in affected patients. Notably, we observed a direct correlation between elevated GANAB levels and increasingly aggressive tumor characteristics, as well as diminished patient outcomes. Functional assays underscored the critical role GANAB plays in driving the proliferation, migration, and invasive capabilities of CRC cells. These findings align with prior research demonstrating GANAB functions as an oncogene in gastric cancer, further supporting its tumorigenic potential across multiple cancer types (Yuan et al., 2014), indicating that GANAB may generally play a role in promoting cancer in gastrointestinal tumors.

Our research shows that GANAB promotes cancer development by activating Wnt/β-catenin signaling pathway. This pathway plays a key role in regulating epithelial-mesenchymal transition (EMT), which is an important process of cancer spread (Lin et al., 2022b; Qin et al., 2017; Wang et al., 2020). In addition, our results also support an increasingly recognized view that carcinogenic pathways such as Wnt will interact with post-transcriptional regulatory systems, thus amplifying their effects (Qu et al., 2022).

After the classical Wnt signaling pathway is activated, it will trigger a series of reactions, and finally stabilize β-catenin so that it can enter the nucleus. Within the cellular nucleus, this protein joins forces with TCF/LEF transcription factors to kick-start the transcription of downstream oncogenes—including heavy hitters like c-Myc and Cyclin D1—which ultimately gives cancer a leg up (Sun et al., 2025; Du et al., 2020; van de Wetering et al., 2002; Yang et al., 2017; Rahmani et al., 2020). What’s more, our investigation revealed a clear connection between GANAB expression and the major players in the Wnt signaling pathway (FZD5, DVL3, CTNNB1, TCF4). Most importantly, after activating Wnt signal with CHIR-99021, the significant dampening of cell proliferation, migration, and invasion observed following GANAB suppression was substantially reversed when the Wnt/β-catenin pathway was blocked. This pattern of findings across multiple experiments solidifies the connection between GANAB’s cancer-promoting activities and its role in triggering the Wnt/β-catenin signaling cascade. A previous study reported that GANAB activates Wnt/β-catenin signaling by promoting Frizzled receptor maturation (Rauscher et al., 2018). Our findings are consistent with this conclusion but reveal a distinct mechanism: we show that GANAB expression is post-transcriptionally upregulated through enhanced mRNA stability, which in turn activates the pathway. Therefore, GANAB supports Wnt/β-catenin signaling through both protein-level and mRNA-level mechanisms.

The most important discovery of our research is that lncRNA FIRRE is a direct post-transcriptional regulator of GANAB. We provided many evidences: the positive correlation of expression level, the physical interaction confirmed by RNA pull-down experiment, and the mRNA decay experiment showed that the combination of FIRRE could enhance the stability of GANAB mRNA. This mechanism is a classic example of how lncRNA can be used as a modular scaffold or “guide” to regulate the fate of target mRNA (Cui et al., 2025; Cai et al., 2025). This specific interaction we verified highlights the accuracy of RNA-RNA recognition in gene regulation. In addition, by mutating the predicted binding site (FIRRE-MUT) in FIRRE, we found that it lost the ability to upregulate GANAB, which confirmed the sequence specificity of this regulation and showed that the therapeutic intervention against this interaction was potential. A formal rescue experiment (FIRRE knockdown plus GANAB overexpression) is still needed to definitively establish the epistatic relationship, and this is a limitation of the current study that will be addressed in future work.

Our study found that lncRNA FIRRE is the upstream regulator of GANAB. Over-expression of FIRRE will stabilize GANAB mRNA and lead to the increase of GANAB protein. Subsequently, Raising the GANAB threshold will bolster the Wnt/β-catenin signaling route, leading to an increase in β-catenin within the cell’s nucleus and consequently amplifying the expression of key downstream elements (such as c-Myc). This series of reactions formed a multi-level regulatory point in CRC. The positive correlation among FIRRE, GANAB, β-catenin and c-Myc in clinical samples further proves the clinical significance of this pathway.

## 5 Conclusion

To put it simply, we have found a regulatory mechanism in CRC: carcinogenic lncRNA FIRRE can directly bind to GANAB mRNA and make it more stable, so the number of GANAB proteins will increase. Elevated GANAB, in turn, potentiates the Wnt/β-catenin pathway through facilitating β-catenin nuclear translocation and the subsequent activation of a pro-tumorigenic transcriptome. The positive correlations observed among FIRRE, GANAB, β-catenin, and c-Myc expression in clinical specimens reinforce the pathological relevance of this axis, which offers a promising targeted strategy for CRC therapy.

## Data Availability

The original contributions presented in the study are included in the article and Supplementary Material, further inquiries can be directed to the corresponding authors.
