# Supplementary material for: Glucosidase GANAB drives colorectal cancer progression through Wnt signaling and is modulated by lncRNA FIRRE
Source: Front Mol Biosci. 2026 Aug 17;13:1820992. doi: 10.3389/fmolb.2026.1820992 (PMC13526586; doi:10.3389/fmolb.2026.1820992)
Supplement: Supplementary file 1 [file DataSheet1.docx]

Supplementary Table 1:

All primer sequences

| Gene | sequence |
| --- | --- |
| GAPDH | F:5’-ATGGTGGTGAAGACGCCAGTA-3’ |
|  | R:5-’GGCACAGTCAAGGCTGAGAATG-3’ |
| FIRRE | F:5’-CTGTGACCTCGCTTCACTTCT-3’ |
|  | R:5’-GTGGCAAAGAGCAGAAGATAGA-3’ |
| GANAB | F:5’ - CCTGTTTGGGAAGATGATGGAC -3’ |
|  | R:5’- GGGTTCCTGTGAGACTAGCATA -3’ |

Supplementary Table 2:

Sequence of siRNA for GANAB knockdown, ASO for FIRRE knockdown

|  | | | sequence |
| --- | --- | --- | --- |
| siGANAB | Si1 | sense | 5’- GCACCAUGCUUGAGCGCUUTT-3’ |
|  |  | antisense | 5’- AAGCGCUCAAGCAUGGUGCTT-3’ |
|  | Si2 | sense | 5’- GGGCUAACAUGUUCAGCUATT-3’ |
|  |  | antisense | 5’- UAGCUGAACAUGUUAGCCCTT-3’ |
|  | Si3 | sense | 5’- GGUAUGACAUUCAAAGCUATT-3’ |
|  |  | antisense | 5’- UAGCUUUGAAUGUCAUACCTT-3’ |
| Target sequence of ASO in FIRRE | | | CTGAAAATGAAGGCCAATAC |

Supplementary Table 3:

Univariate analyses of the factors correlated with Overall survival of cancer patients

| Variables | Univariate analysis | | |
| --- | --- | --- | --- |
|  | HR | 95%CI | |
|  |  | Lower | Upper |
| GANAB expression | 1.959* | 1.021 | 3.757 |
| Sex | 0.904 | 0.478 | 1.709 |
| Age | 0.742 | 0.39 | 1.413 |
| Grade stage | 2.7* | 1.271 | 5.736 |
| Tumor size | 1.341 | 0.691 | 2.603 |
| T stage | 3.281*** | 1.723 | 6.25 |
| N stage | 3.5*** | 1.83 | 6.692 |
| M stage | 4.951** | 1.843 | 13.303 |
| TNM stage | 4.163*** | 2.141 | 8.094 |

* p<0.05, **p<0.01, ***p<0.001, ****p<0.0001.

Supplementary Table 4:

Multivariate analyses of the factors correlated with Overall survival of cancer patients

| Variables | Univariate analysis | | | |
| --- | --- | --- | --- | --- |
|  | HR | 95%CI | | P value |
|  |  | Lower | Upper |  |
| GANAB expression | 1.28 | 0.64 | 2.56 | 0.485 |
| Grade stage | 1.26 | 0.55 | 2.9 | 0.586 |
| T stage | 1.85 | 0.87 | 3.91 | 0.109 |
| N stage | 0.71 | 0.11 | 4.6 | 0.718 |
| M stage | 1.79 | 0.5 | 6.38 | 0.372 |
| TNM stage | 3.64 | 0.47 | 28.16 | 0.215 |

Supplementary Figure 1:

GANAB promotes proliferation and invasion of colorectal cancer cells. A and B, The CCK-8 assay was implemented to detect the effect of overexpression of GANAB on the proliferation of HT29 and LOVO cells. Results are shown as the mean ± SD. C. The effect of overexpression of GANAB on the migration of HT29 and LOVO cells was analyzed by Transwell assay. Results are shown as the mean ± SD. D. The effect of overexpression of GANAB on the invasion of HT29 and LOVO cells was analyzed by wound-healing assay. The experiments were repeated three times. **p<0.01, ***p<0.001, ****p<0.0001.
